# Community perception of malaria in a vulnerable municipality in the Colombian Pacific

**DOI:** 10.1186/s12936-020-03404-4

**Published:** 2020-09-21

**Authors:** Carol Yovanna Rosero, Gloria Isabel Jaramillo, Franco Andrés Montenegro, César García, Arelis Alexandra Coral

**Affiliations:** 1 Interdisciplinary Research Group in Health and Disease, (Grupo Interdisciplinario de, Investigación en Salud-Enfermedad-GIISE), Universidad Cooperativa de Colombia, Medicine Faculty, San Juan De Pasto, Nariño, Colombia; 2 Research Group of Villavicencio (Grupo de Investigación de Villavicencio-GRIVI), Medicine Faculty, Universidad Cooperativa de Colombia, Villavicencio, Colombia; 3 Master’s Degree in Infections and Health in the Tropics, University of Nacional Colombia, Bogotá, Colombia

**Keywords:** Malaria, Community, Knowledge, Attitudes, Practice, Colombia

## Abstract

**Background:**

Malaria primarily affects populations living in poor socioeconomic conditions, with limited access to basic services, deteriorating environmental conditions, and barriers to accessing health services. Control programmes are designed without participation from the communities involved, ignoring local knowledge and sociopolitical and cultural dynamics surrounding their main health problems, which implies imposing decontextualized control measures that reduce coverage and the impact of interventions. The objective of this study was to determine the community perception of malaria in the municipality of Olaya Herrera in the Colombian Pacific.

**Methods:**

A 41-question survey on knowledge, attitudes, and practices (KAP) related to malaria, the perception of actions by the Department of Health, and access to the health services network was conducted.

**Results:**

A total of 134 adults were surveyed, in whose households a total of 671 people lived. According to the survey data, about 80% of the household members included teenagers and children, out of which 61% had malaria at one time, and for 75.3%, this disease is a persistent problem. In spite of this, 57.2% of people who fell ill due to malaria were never visited by health personnel for a follow up. This population claimed that responsibility for who should prevent the disease is shared between each person and the Department of Health. However, personal actions were focused on using mosquito nets, ignoring other important practices to prevent bites. Despite campaigns by the Department of Health, 11.9% of respondents did not know how malaria was transmitted, and 8.96% thought it was transmitted through water. Also, 43.5% said that the Department of Health did not do any work to control malaria and 16% did not know if any action was taken.

**Conclusions:**

In spite of the knowledge about malaria and the efforts of the Department of Health to prevent it, the community actions do not seem to be consistent with this knowledge, as the number of cases of malaria is still high in the area.

## Background

Malaria is one of the parasitic diseases with the highest rate of morbidity and mortality worldwide [1], with more than 50 million cases per year and with affliction rates that reach 64 every thousand inhabitants. Controlling this disease is costly and epidemics have a negative impact on the socioeconomic development of the affected areas and on the population’s vulnerability [2]. Even though 12 of the 18 endemic countries in the Americas region are aiming to achieve a ≥ 40% reduction in case incidence by 2020, cases in Colombia doubled between 2015 and 2016, making it a continued event of interest in public health [3, 4]. According to the National Institute of Health [4], Colombia is an endemic country for this disease, whose foci of infection are concentrated mainly in Chocó, Nariño, Córdoba, Antioquia, Guainía, Amazonas, Bolívar, and Vichada, where 87.9% of the cases are recorded. The and illegal mining areas [5].

During the first 29 epidemiological weeks of 2018 in Colombia, 33,501 cases of malaria were reported to the Public Health Surveillance System (Sivigila), which places Colombia at 12%, with the third highest incidence of malaria, after Brazil (42%) and Venezuela (18%), which have the highest burden of disease in America [6]. Within the country, Chocó, Nariño, Córdoba, Antioquia, National Institute of Health established that 8% of cases occur in mining settlements and areas with illicit crops concentrated mainly in Nariño, Chocó, and Antioquia (18.7% of cases). Many malaria cases in the country develop in areas beyond the health sector’s reach, such as in subnormal settlements Guainía, Amazonas, Bolívar, and Cauca are the departments with the highest risk of contracting the disease [7].

The maintained high rates of malaria and the recurrence of its endemic–epidemic profile in the population is explained, as approximately 85% of Colombia’s rural territory is located less than 1,600 meters above sea level and has climatic, geographic, and epidemiological conditions suitable for the transmission of the disease [4]. Moreover, they are closely related to situations of social and economic vulnerability faced by those living in rural areas on the Pacific coast, making it a perfect location for malaria transmission [8]. These populations have had an intense, prolonged coexistence with malaria. For this reason, they have developed unique conceptions about the disease, the methods of dealing with it, and the health services and professionals working in this field.

As malaria is a multicausal disease, new approaches and methods to control it must integrate individual participation, community empowerment and institutional leadership that strengthens the capacity of local responses to achieve sustainability of actions with an emphasis on promotion and prevention [9]. Historically in Colombia, the programmes to control and eradicate the disease have been characterized by their vertical nature with low community participation [10, 11].

Therefore, faced with the challenges created by social determinants in endemic malaria regions (armed conflicts, displacement, access barriers, deforestation, among others), it is necessary to rethink community participation in the scenarios applied. The rural area represents 30% of the population, and particularly, in Nariño, it represents 51%. The rural area has been historically forgotten in discourse and intervention, and when there are interventions, it is assumed that those who do not live in the countryside have the solutions. Therefore, an approach is needed to the social construction of health and disease in the rural population, their collective imagination, needs, and life projects, which differ from the viewpoints of the city dweller [12].

Their use of resources, the perception of life cycle, and their productive role as a family are constructed differently, which is perhaps why interventions have had little positive effect [13]. Thus, it is necessary to include the community’s voices represented in the discussions and tasks facing the disease, its prevention and its treatment. Therefore, this study aims to provide a community and institutional view on these elements in relation to the disease and the vector.

## Methods

**Fig. 1:**
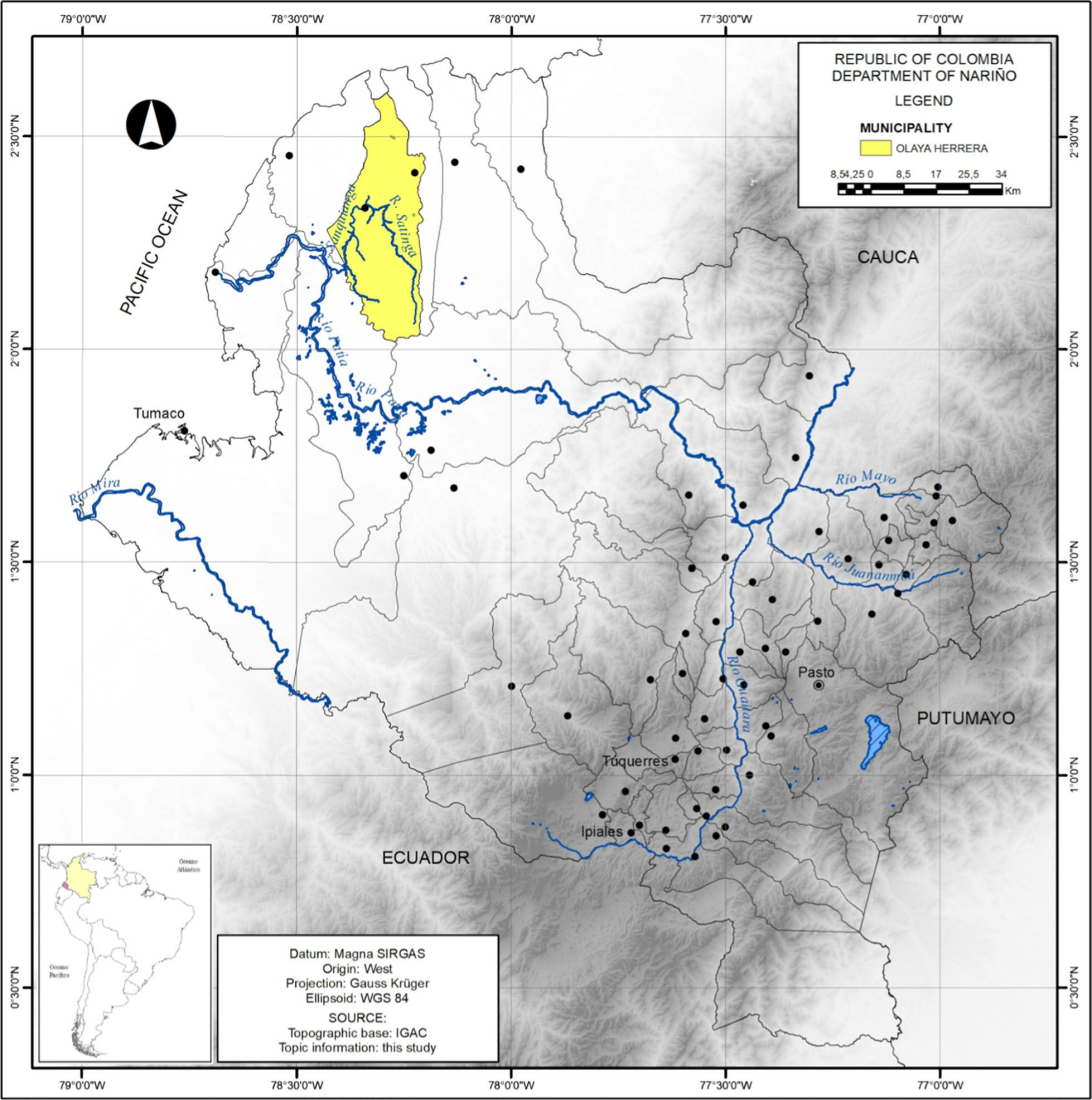
Location of the municipality of Olaya Herrera (Nariño–Colombia). Source: The authors

A descriptive observational study was conducted using a knowledge, attitudes, and practices (KAP) survey method in the municipality of Olaya Herrera, located northwest of the Department of Nariño and in the Southwest of Colombia in the Pacific Plain region. The municipal seat of Olaya Herrera is Bocas de Satinga, located at the geographic coordinates of 2°20′52.97″ N and 78°19′31.27″ W, in the northern sector of the municipal territory, where the Satinga and Sanquianga rivers meet (Fig. 1).

The “Survey on Knowledge, Attitudes and Practices (KAP) on the approach to malaria in indigenous communities” [13] was conducted with some modifications for its application in the community of Olaya Herrera. It consisted of 41 questions within the variables on basic, general data, KAP about the disease, and perception of health systems, applied to the municipality’s inhabitants (Additional file 1).

To conduct the surveys, the city map was divided into neighborhoods, from which a random starting point was chosen. The KAP survey was conducted in the first home that was visited, where a resident agreed to participate in the study. The next house visited was the third counting from the previous one, in the clockwise direction, and so on. If an uninhabited house or a vacant lot was encountered, the next house was checked. At least 60% of the urban area in the municipality was covered before the sample was completed. The inclusion criteria were: people of legal age, municipality residents for at least 2 years, lived in the house surveyed, and prior informed consent given.

The sample size was calculated with a confidence level of 95%, a margin of error of 5%, and an expected malaria amount of 0.05, according to the data reported by the Department of Health of the municipality of Olaya Herrera for the year 2013. Based on these parameters, a minimum sample size of 73 surveys was estimated for residents of the municipality’s urban area.

$$n = \frac{{Z^{2} *p*\left( {1 - p} \right)}}{{e^{2} }}$$

An initial descriptive analysis was made on the KAP survey variables based on frequencies and measures of central tendency depending on the nature of each variable. For the intersection of variables, non-parametric statistics were found by analyzing *X*^*2*^ and Kruskal–Wallis. The statistical programs EpiInfo 7.0 and SPSS Statistics V24.0 were used.

Additionally, a bivariate analysis was conducted with a focus on social determinants of health. From the data, the descriptive sociodemographic variables were considered as analysis factors (risk/protectors) and as an effect of having contracted malaria or not throughout their life. The data were analysed in 2 × 2 contingency tables and OR incidence was calculated, seeking to delimit the variables most likely associated with having the disease or not, from the social determinants. A value of p < 0.05 was considered significant in the X^2^ tests and values different than 1 in IC95%.

## Results

**Fig. 2:**
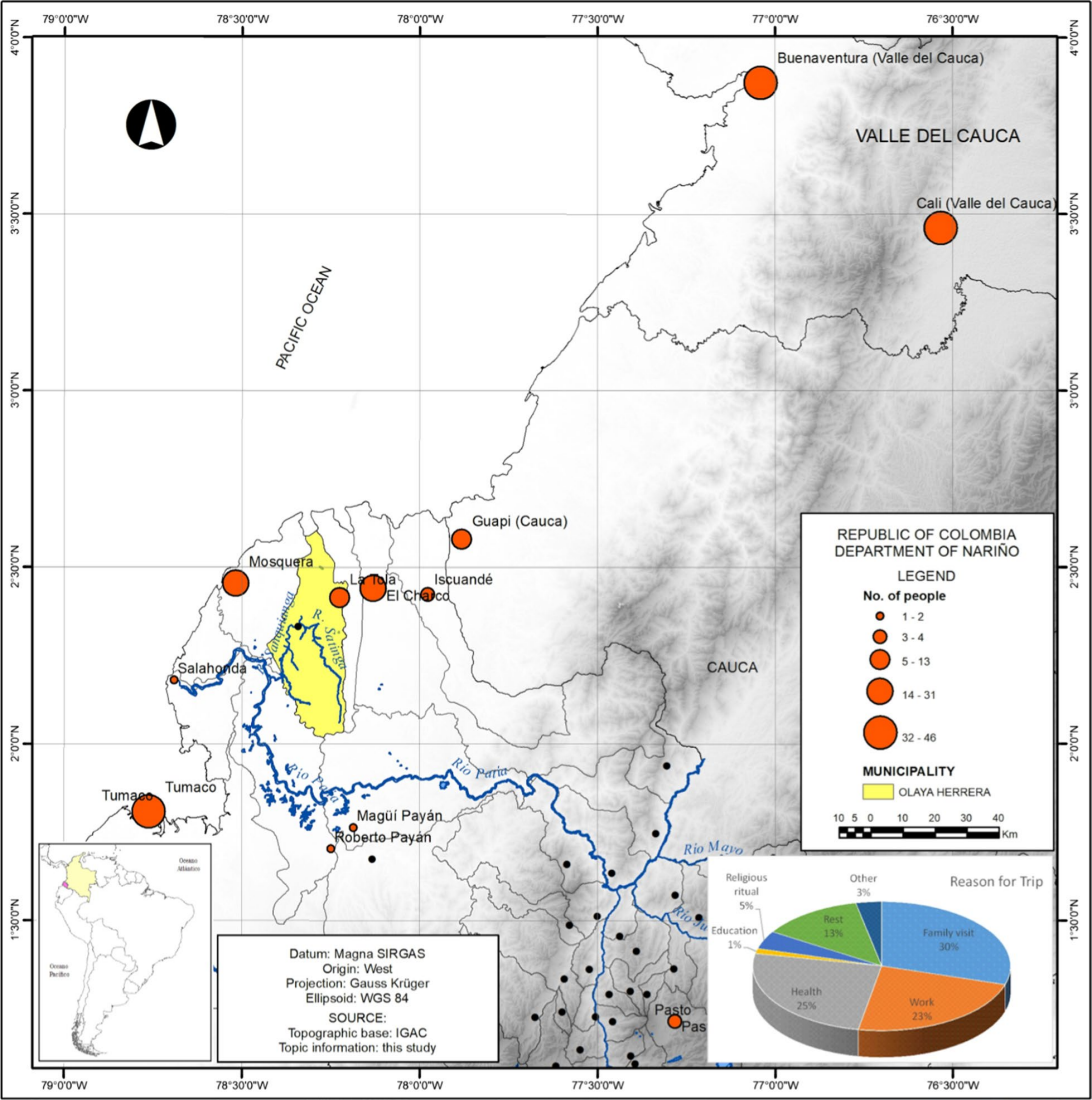
Municipalities frequented by the population of Olaya Herrera (Nariño–Colombia) Source: The authors

Visits were made to the municipality during the months of February and April of 2018. The KAP survey was conducted among 134 adults in the municipality: 34.6% were men and 65.4% were women. The average age of the respondents was 39.79 years (SD ± 15.70 years). The oldest person surveyed was 96 years old. This population, on average, has lived in the municipality of Olaya Herrera for 23.26 years (SD ± 16.61 years), with a minimum of two years and a maximum of 92 years (Table 1). In general terms, it is not a mobile population, and its movement outside the municipality is limited to nearby municipalities such as Tumaco (19.83%), Cali (18.14%), Buenaventura (16.88%), Mosquera (13.08%), and El Charco (10.54%), which are municipal and departmental capitals. Trips are made primarily for family visits (34.33%, IC95% 26.35–43.02), work (26.87%, IC95% 19.58–35.20) or health (29.10%, IC95% 21.58–37.57) (Fig. 2).

**Table 1:** Sociodemographic characteristics, living conditions, knowledge, attitudes and practices of the population of Olaya Herrera Colombia

		Average	±SD	
Time living in the municipality (years)		23.26	16.61	

		n	%	IC 95%
Sociodemographic characteristics				
Sex				
	Female	87	65.41	56.68–73.44
	Male	46	34.59	26.56–43.32
Marital status				
	Married	18	13.43	8.16–20.40
	Divorced	6	4.48	1.66–9.49
	Single	56	41.79	33.33–50.62
	Domestic partnership	51	38.06	29.82–46.84
	Widow(er)	3	2.24	0.46–6.40
Health affiliation				
	Contributory	9	6.72	3.12–12.37
	Special	1	0.75	0.02–4.09
	None	5	3.73	1.22–8.49
	Subsidized	119	88.81	82.21–93.60
Education level				
	Illiterate	13	9.85	5.35–16.25
	Primary	40	30.3	22.61–38.90
	Secondary	53	40.15	31.72–49.04
	Technical	16	12.12	7.09–18.94
	University	10	7.58	3.69–13.49
Occupation				
	Farming	6	4.48	1.66–9.49
	Craftwork	1	0.75	0.02–4.09
	Unemployed	9	6.72	3.12–12.37
	Public employee	8	5.97	2.61–11.42
	Private company	16	11.94	6.98–18.67
	Livestock/Agriculture	5	3.73	1.22–8.49
	Home	26	19.4	13.08–27.12
	Independent	55	41.04	32.63–49.87
	Laborer	6	4.48	1.66–9.49
	Worker	2	1.49	0.18–5.29
Living conditions of houses				
Roof type				
	Cement	4	3.03	0.83–7.58
	Vegetation or plant	2	1.52	0.18–5.37
	Zinc or laminate	124	93.94	88.41–97.35
	Other	2	1.52	0.18–5.37
Wall type				
	Wood	119	89.47	82.97–94.12
	Spackled or painted	8	6.02	2.63–11.51
	Only cement	4	3.01	0.83–7.52
	Other	2	1.5	0.18–5.33
Floor type				
	Tile	8	5.97	2.61–11.42
	Cement	9	6.72	3.12–12.37
	Other	117	87.31	80.47–92.43
Electricity		131	97.76	93.60–99.54
Garbage disposal		88	65.67	56.98–73.65
Running water		45	33.58	25.66–42.25
Water well		44	32.84	24.97–41.47
River/ravine		86	64.18	55.44–72.27
Toilet		36	26.87	19.58–35.20
Septic tank		89	66.42	57.75–74.34
Sewage system		11	8.21	4.17–14.21
How many people live at home				
	n	Average	±SD	%
Children	167	1.25	1.30	24.89
Teenagers	374	2.79	1.46	55.74
Adults	22	0.17	0.48	3.28
Eldery	108	0.82	1.29	16.10
How many times do you travel to other municipalities				
		Average	±SD	
		4.02	6.7	
For how long do you stay out (days)				
		14.42	21.42	
Why do you travel out of your home				
		n	%	IC 95%
	They are close	1	0.75	0.02–4.09
	Family visit	46	34.33	26.35–43.02
	Work	36	26.87	19.58 –35.20
	Health	39	29.1	21.58–37.57
	Education	2	1.49	0.18–5.29
	Religion	7	5.22	2.13–10.47
	Break	20	14.93	9.36–22.11
	Other	5	3.73	1.22–8.49
Knowledge				
Have you had malaria				
	Yes	81	60.9	52.07–69.24
	No	51	38.35	30.05–47.17
	Doesn’t know	1	0.75	0.02–4.12
Any family member has had malaria more than once				
	Yes	25	18.66	12.45–26.30
	No	105	78.36	70.42–85.00
Malaria is a health problem for you				
	Yes	101	75.37	67.19–82.40
	No	23	17.16	11.20–24.63
	Doesn’t know	10	7.46	3.64–13.30
Who should prevent malaria				
	Each person	54	40.3	31.92–49.11
	Community	4	2.99	0.82–7.47
	Family	3	2.24	0.46–6.40
	Doesn’t know	20	14.93	9.36–22.11
	Other	2	1.49	0.18–5.29
	Health Secretary	51	38.06	29.82–46.84
How do we get malaria				
	Water	12	8.96	4.71–15.12
	Contaminated food	1	0.75	0.02–4.09
	Doesn’t know	16	11.94	6.98–18.67
	Other	1	0.75	0.02–4.09
	Parasites inside mosquitos	1	0.75	0.02–4.09
	Bite of any mosquito	93	69.4	60.86–77.07
	Bite of anopheles mosquito	10	7.46	3.64–13.30
What are the symptoms of malaria				
	High fever	102	76.12	67.99–83.06
	Headache	106	79.1	71.24–85.64
	Muscle and bone pain	42	31.34	23.61–39.92
	Chill	84	62.69	53.92–70.88
	Weakness and tiredness	20	14.93	9.36–22.11
	Vomiting and diarrhea	45	33.58	25.66–42.25
	Doesn’t know	18	13.43	8.16–20.40
	Other	12	8.96	4.71–15.12
Attitude				
How malaria is cured				
	Going to the hospital	9	6.77	3.14–12.46
	Going to the health center	26	19.55	13.19 –27.32
	Other	7	5.26	2.14–10.54
	Taking the treatment formulated by the doctor	86	64.66	55.91–72.75
	Taking traditional medicine	3	2.26	0.47–6.45
	Taking other medicine	2	1.5	0.18–5.33
How many known people have died from malaria				
	0	106	80.3	72.49–86.71
	1	12	9.09	4.79–15.34
	2	5	3.79	1.24–8.62
	3	1	0.76	0.02–4.15
	4	2	1.52	0.18–5.37
	10	4	3.03	0.83–7.58
	15	1	0.76	0.02–4.15
	30	1	0.76	0.02–4.15
Death was confirmed by a doctor				
	Yes	12	10.62	5.61–17.82
	No	15	13.27	7.62–20.95
	Doesn’t know	86	76.11	67.17–83.63
What test to diagnose malaria has been done				
	Thick blood	37	27.61	20.24–36.00
	Blood sample	49	36.57	28.42–45.32
	None	42	31.34	23.61–39.92
	Other	2	1.49	0.18–5.29
	Doesn´t know the name	4	2.99	0.82–7.47
You take all the formulated medicine				
	Yes	124	96.12	91.19–98.73
	No	2	1.55	0.19–5.49
	Doesn’t know	3	2.33	0.48–6.65
Agrees with fumigation at home for vector control				
	Yes	118	96.72	91.82–99.10
	No	3	2.46	0.51–7.02
	Doesn’t know	1	0.82	0.02–4.48
If you have had malaria, some health personnel have visited you				
	Yes	19	14.5	8.96–21.72
	No	75	57.25	48.32–56.85
	Doesn’t know	2	1.53	0.19–5.41
	Have never had malaria	35	26.72	19.37–35.15
Know someone who cures malaria and is not a doctor				
	Yes	7	5.3	2.16–10.62
	No	100	75.76	67.53–82.79
	Doesn’t know	25	18.94	12.65–26.68
Practice				
How do you take care not to get sick from malaria				
	Filling the trenches with earth	28	209	14.36–28.76
	Organizing cleaning days with the community	5	3.73	1.22–8.49
	Draining stains	26	19.4	13.08–27.12
	Drilling objects that may contain water	6	4.48	1.66–9.49
	Using wire mesh on windows	1	0.75	0.02–4.09
	Using mosquito nets	110	82.09	74.53–88.17
	Using repellents	20	14.93	9.36–22.11
	Personal protection (clothing impregnated)	3	2.24	0.46–6.40
	Fumigation	19	14.18	8.76–21.25
	Using plastic nets on doors/windows	0	0	0
	Doesn’t know	13	9.7	5.2–16.02
	Does nothing	6	4.48	1.66–9.49
Health service perception				
Receives good health care in the medical consultation				
	Yes	68	52.71	43.74–61.56
	No	50	38.76	30.31–47.73
	Doesn’t know	11	8.53	4.33–14.75
The health secretary carries out community activities against malaria				
	Yes	53	40.46	13.98–49.38
	No	57	43.51	34.88–52.45
	Doesn’t know	21	16.03	10.21–23.45
The health secretary carries out education days against malaria				
	Yes	34	25.56	18.40–33.85
	No	69	51.88	43.05–60.62
	Doesn’t know	30	22.56	15.77–30.61
What health service do you go to when you get sick				
	Health center	103	78.03	70.00–84.77
	Hospital	7	5.3	2.16 –10.62
	None	2	1.52	0.18–5.37
	Other	20	15.15	9.51 –22.43

The majority of respondents were single (41.8%) or in a domestic partnership (38.06%). Almost 90% of this population belongs to the subsidized system, a mechanism through which the poorest population, without the ability to pay, has access to health service through a subsidy offered by the state, and an important 3.8% does not have any affiliation to the health system. Only 20% of this population has higher education studies (either technical or professional), and 10% declared themselves illiterate (Table 1).

When analysing the family environment of the respondents, the majority of their household members were adolescents (55.73%) and children (24.89%) (Table 1), a condition that is reflected with a childhood index of 38.6 under 15 for every 100 people. Similarly, there was a significant percentage of older adults (16.09%); however, the old age index for the population is 3.56 inhabitants over 65 for every 100 people [14].

Living conditions and basic needs vary greatly in the municipality. Only 33.6% of the population has running water and 8.2% has a sewage system. Only 65% of the population has garbage collection (Table 1).

In this municipality, 61% (IC95% 52.07–69.24) of the respondents have contracted malaria, which represents a significant percentage of patients in the population. Additionally, 18.66% (IC95% 12.45–26.30) of the respondents said that some member of their family or themselves had contracted malaria more than once in the same year. This is directly related to the fact that 75.37% (IC95% 67.19–82.40) consider this disease a problem both for themselves and their families (Table 1).

As for the question of who should prevent malaria, the population is aware that it is not a task that should be delegated only to government agencies such as the Department of Health, but that personal participation is also very important to manage and prevent malaria. This is corroborated later with the question regarding what they do to avoid getting sick from malaria. The majority of respondents said that they use mosquito nets; as a result, personal management is used the most, and once again, community options are completed the least. Approximately 38.7% of the respondents claim that they have not received good health care from health officials after being sick with malaria, with 90.98% of the respondents with malaria having gone to health centres and following treatments recommended by the doctor. Out of the total respondents, 43% say that the Department of Health does not make any effort to reduce malaria in the municipality and 51% say that there are no educational campaigns on malaria (Table 1).

In relation to disease transmission, 69.4% recognize a mosquito as its transmitting vector, despite not knowing its specific name; however, only 7.46% fully know the vector including its specific name. Due to the high percentage of the surveyed population having contracted malaria at some point in their life, there is also knowledge about the symptoms of malaria; noting headaches (79.10%), high fevers (76.12%), chills (62.69%), vomiting and diarrhea (33.58%), and muscle and bone ache (31.34%) as specific symptoms of this disease (Table 1).

In relation to the questions related to the identification of the attitudes of the respondents (Table 1), 64.66% take the treatment formulated by the doctor, 10.62% of them know someone who died of malaria confirmed by a doctor, 36.57% reported that they did the thick gout test to confirm the diagnosis of malaria. 96.72% of the participants agree with home fumigation for vector control. Regarding the practices implemented, 78.03% of the referred respondents will go to the health center when they are sick and 15.05% will have another type of medium.

In the bivariate analysis with a focus on social determinants of health, we observed that the survey suggests the risk factors of males, being single or separated, staying at home, being unemployed or having informal employment, having houses with zinc or laminate walls, dirt floor or other, and the absence of services such as garbage collection and a sewer system. However, the analysis did not yield a statistically significant *p* value, but contributed and confirmed that unsatisfied basic needs are a health risk factor for these communities and especially for diseases transmitted by vectors (Table 2).

**Table 2:** Bivariate Analysis of Social Determinants of Malaria in Olaya Herrera (Nariño)

Variable	Risk factor	OR	IC95%	*P*
Sex M/F	Male	1.44	0.67–3.05	0.34
Age < 40 > 40	< 40	0.77	0.38–1.57	0.47
Marital status	Single/other	1.40	0.7–2.81	0.34
SS System	Uninsured/subsidized	1.04	0.27–3.88	0.95
Education level	Illiterate/primary	0.67	0.33–1.37	0.28
Type of employment	Home/informal	1.21	0.56–2.59	0.62
Home wall type	Other/zinc laminate	2.33	0.69–4.04	0.16
Home floor type	Dirt/other	1.45	0.52–4.04	0.47
Garbage collection	None	1.32	0.63–2.77	0.46
Running water	None	0.97	0.46–2.03	0.93
Sewage system	None	1.56	0.09–2.57	0.75

## Discussion

KAP surveys allow us to understand the context of quantitative research numbers and epidemiological indexes [12, 15]. This study was conducted to identify the KAP related to malaria in a vulnerable municipality of Colombia, a community with a long history of social segregation and poverty.

The sociodemographic characteristics showed that in terms of house structure, zinc roofs followed by wood walls were the most prevalent materials types of houses. Similar results were observed in houses of communities in Quibdo (Colombia) [15], in Swaziland (Africa) [16] and Chiapas (México) [17], showing some environmental characteristics similar to other endemic malaria countries.

There remain some equity issues regarding education, such as 9.85% of the surveyed people being illiterate, mostly women (69.2%). However, these results were lower than those observed in other studies in which the level of the population with no education is higher, 16.2% in Swaziland (Africa) [16], 23.6% in Muleba (Africa) [18], 29.2% in Rural Northwest Tanzania (Africa) [19], and 16.6% in Chiapas (México) [17].

When analyzing the family environment of the respondents, the majority of their household members were adolescents (55.73%) and children (24.89%), a condition that is reflected with a childhood index of 38.6 under 15 years old for every 100 people. Similarly, there was a significant percentage of eldery (16.09%); however, the old age index for the population is 3.56 people over 65 years old for every 100 people [14]. This could affect the municipality’s economy, resulting in a small workforce or people forced to work from very early to very advanced ages.

Public services are absent or deficient, especially drinking water supplies (33.58%) and garbage (65.67%) and sewage disposal (8.21%). These findings are similar to those found in Calcutta in India [20].

All those points above listed, combine with focal points of inequality in the social structure, such as the low-income social class with few basic needs met, socioeconomic position, gender with great inequalities for women and an ethnic group that marks a whole territory of inequality, where Afro-Colombian mixes with the high rurality full of oversight and poverty [15].

The knowledge of this community about malaria, evidenced that they know that malaria is a disease transmitted by a mosquito and that the responsibility to prevent it falls not only on government entities such as the Department of Health but also on every individual and the community. These results are in line with studies conducted in Tumaco and Buenaventura, nearby municipalities similar to Olaya Herrera in culture and ethnicity, where between 79.2% and 86.9% of the respondents knew that malaria transmission occurs through the bite of an infected mosquito [21]. These levels of knowledge are not only seen in Colombian communities. In other populations like in the northern coast of Ecuador between 50 and 75% of the surveyed people in the communities declared to know how malaria is transmitted and 90–100% knew that a mosquito is responsible for malaria transmission [22]. The majority of the respondents also associated mosquito bites with malaria transmission, in Zambia (Africa) [23], Swaziland (Africa) [16], in Muleba (Africa) [18]. This leads us to believe that the problem lies not in what populations know about malaria, but in the disconnection between them and the disease control plans taught by Departments of Health and other government entities [24, 25].

The knowledge of signs and symptoms showed that over 76% of the respondents identified fever, headache, and chills as the most common ones. This is in line with the observations of most studies in endemic settings from Colombia, as in Antioquia and Choco where 80% of knowledge was found on the main symptoms of malaria [26], and in other contexts like Swaziland (Africa) [16], Muleba (Africa) [18] and Rural Northwest Tanzania (Africa) [19].

The population’s attitudes about malaria control observed in this study are similar to the population of Chiapas (Mexico) [17], more than 96% of the respondents agreed with the use of insecticides at home as a strategy to protect their homes from mosquito bites in the future.

Regarding practices to prevent malaria, the health system performs community education, and is making efforts to spread awareness regarding the risks posed by vectors; the population accepts these trainings and the level of knowledge is acceptable. However, such methods will not resolve problems regarding the environment, homes located near vegetation and the river, scarcity of drinking water, and the lack of garbage disposal, which are breeding grounds for high-risk conditions for the population’s health. There is a health center with first level basic care focused on treatment and rehabilitation with few resources allocated to activities promoting health and preventing diseases. Accessing levels of greater complexity is very difficult due to economic, geographic, and communication barriers, without dismissing the violent actors that are part of daily life.

The KAP survey results suggest that this prolonged process of coexisting with malaria and prevention and control campaigns could be reflected in the homogeneity of the responses among all the participants regarding their perception of the disease as one of the main health problems (75.37%). Some of the field observations showed the lack of control measures and stagnant water, and the use of insecticides and mosquito nets as the most important control measures in this community, being the same reported by Fernández-Niño (2014) [27] and Osorio (2006) [28] in similar populations like the northern coast of Ecuador [22], in Iquitos (Perú) [29], Chiapas (México) [17], and others populations like Swaziland (Africa) [16], in Rural Northwest Tanzania (Africa) [19].

The perception of the population about preventive activities to control malaria in Olaya Herrera is not the best one; because more than 40% said that the Health Department doesn’t do any control activities. Similarly, a low perception of the benefit of preventive activities has been reported as a result of the lack of concerted action between the health services and the community en Lima–Perú [30].

## Conclusions

In general, the population knows what malaria is, its symptoms, how it is transmitted, and how it can be prevented, which could lead to the conclusion that the Department of Health has organized information campaigns in this regard. However, community actions do not seem to be consistent with this knowledge, as the number of cases of malaria is still high and environmental conditions are still favorable for vector breeding. The malaria control programs carried out by the government entities would be focused on effective interventions to address malaria specific risk factors. However, to ensure a health promoting environment in which these populations live and can then practice the appreciate a malaria prevention behaviours, probably a broader a primary care strategy involving a family and community approach is required.

## Supplementary information

**Additional file 1.** KAP survey made to the population of Olaya Herrera—Colombia.

## Data Availability

The datasets used and/or analysed during the current study are available from the corresponding author on reasonable request.
