# Supplementary material for: Community perception of malaria in a vulnerable municipality in the Colombian Pacific
Source: Malar J. 2020 Sep 21;19:343. doi: 10.1186/s12936-020-03404-4 (PMC7507275; doi:10.1186/s12936-020-03404-4)
Supplement: Supplementary file 1 — Additional file 1. KAP survey made to the population of Olaya Herrera—Colombia. [file 12936_2020_3404_MOESM1_ESM.docx]

**Annex 1.**

KAP SURVEY

Lattitude: (to be filled by the researcher)

Longitude: (to be filled by the researcher)

Date: _____________________

Name: ____________________

Department: ________________

Municipality: ________________

Neighborhood: ______________

Economic level condition (1-6 only for colombian municipalities):

1. **General data**

1. How long have you lived in this municipality (years):

2. How many people live in your house:

Children:

Teenagers

Adults

Eldery

3. Sex: M F

4. Marital status : Married

Divorced

Single

Domestic Partnership

Widow(er)

5. Health affiliation: Contributory

Special

Subsidized

None

6. Education level: Illiterate

Primary

Secondary

Technical

University

7. Occupation: Farming

Craftwork

Unemployed

Public employee

Private company

Livestock/Agriculture

Home

Independent

1. **Living conditions of houses**

8. Roof type:

Cement

Vegetation or plant

Zinc or laminate

Other

9. Wall type: Wood

Spackñed or painted

Only cement

Other

10. Floor type: Tile

Cement

Other

11. Your house has: Electricity

Garbage collection

Running water

Water well

River/ravine

Toilet

Septic tank

Sewage system

12. How many times per year do you travel to other municipalites:

13. For how long do you stay out (days):

14. Why do you travel to those places: They are close

Family visit

Work

Health

Education

Religion

Break

Other

1. **Knowledge**

15. Have you had malaria: Yes

No

Doesn´t know

16. Any family member has had malaria more than once (number)

17. Considers malaria a health problem for you and your family: Yes

No

Doesn´t know

18. Who should prevent malaria: Each person

Community

Familiy

Health Secretary

Doesn´t know

Other

19. How do we get malaria: Water

Contaminated food

Parasites inside mosquitos

Bite of any mosquito

Bite of Anopheles mosquito

Other

Doesn´t know

20. What are the sympoms of malaria: High fever

Headache

Muscle and bone pain

Chill

Weakness and tiredness

Vomiting and diarrhea

Doesn´t know

Other

21. How malaria is cured: Going to the hospital

Going to the health center

Taking the treatment formulated by the doctor

Taking traditional medicine

Taking other medicine

Other

22. How many known people have died from malaria

23.The death was confirmed by a doctor: Yes

No

Doesn´t know

24. What test have been done to diagnose malaria: Thick blood

Blood sample

None

Other

Doesn´t know the name

1. **Attitude**

25. Do you take all the formulated medicine by the doctor: Yes

No

Doesn´t know

26. Do you agree with the fumigation to houses for vector control: Yes

No

Doesn´t know

27. If you have had malaria, some health personnel have visited you at home:

Yes

No

Doesn´t know

28. Do you know someone who cures malaria ans is not a doctor: Yes

No

Doesn´t know

1. **Practices**

29. How do you take care not to get sick from malaria:

Filling the trenches with earth

Organizing cleaning days with the community

Draining stains

Drilling objects that may contain water

Using wire mesh on windows

Using mosquito nets

Using repellents

Personal protection (clothing impregnated)

Fumigation

Using plastic nets on doors/windows

Doesn´t know

Does nothing

1. **Perceptions of the actions of Health Secretary**

30. Do you recieve good health care when you have malaria: Yes

No

Doesn´t know

31. The health secretary carries out community work against malaria: Yes

No

Doesn´t know

32. The health secretary carries out education days against malaria: Yes

No

Doesn´t know

33. What health service do you go to when you get sick: Health center

Hospital

None

Other
